# Direct evidence for dendritic spine compensation and regeneration in Alzheimer's disease models

**DOI:** 10.1002/alz.70829

**Published:** 2025-10-23

**Authors:** Nishita Bhembre, Zoran Boskovic, Jessica Louise Willshaw, Tim Castello‐Waldow, Calum Bonthron, Annalisa Paolino, Patricio Opazo

**Affiliations:** ^1^ Clem Jones Centre for Ageing Dementia Research Queensland Brain Institute The University of Queensland Brisbane Queensland Australia; ^2^ School of Psychology and Public Health La Trobe University Melbourne Victoria Australia; ^3^ UK Dementia Research Institute Institute for Neuroscience and Cardiovascular Research University of Edinburgh Edinburgh UK; ^4^ School of Biomedical Sciences Faculty of Medicine The University of Queensland Brisbane Queensland Australia

**Keywords:** Alzheimer's disease, cognitive reserve, cognitive resilience, dendritic spines, homeostatic plasticity, in vivo two‐photon imaging, synaptic loss, synaptic plasticity

## Abstract

**INTRODUCTION:**

Dendritic spine loss in Alzheimer's disease (AD) strongly correlates with cognitive decline, whereas spine preservation is associated with cognitive resilience. Yet, whether and how neurons compensate for spine loss in AD remains largely unknown.

**METHODS:**

We developed a chromophore‐assisted light inactivation (CALI) strategy to selectively eliminate dendritic spines to model this key feature of AD. Two‐photon microscopy was used to monitor the structural plasticity of spines over time after spine elimination. Validation experiments were conducted in amyloid beta (Aβ)–driven models of synapse loss, including APP/PS1 mice and intracortical delivery of oligomeric Aβ.

**RESULTS:**

We discovered that dendritic spine elimination—induced either artificially or in Aβ models—triggers a two‐stage compensatory response: rapid enlargement of remaining spines followed by delayed spine regeneration.

**DISCUSSION:**

These findings provide direct evidence that neurons retain an intrinsic capacity to reverse early synaptic loss in AD, potentially contributing to cognitive resilience.

**Highlights:**

- We developed a targeted optogenetic tool to selectively eliminate individual dendritic spines in live neurons, both in vitro and in vivo.
- We discovered a two‐stage compensatory response to spine loss: rapid enlargement of surviving spines followed by delayed regeneration.
- We showed that the compensatory enlargement of dendritic spines depends on N‐methyl‐D‐aspartate receptor activation and protein synthesis.
- We validated across multiple Alzheimer's disease models, demonstrating that similar compensatory plasticity occurs after amyloid beta oligomer–induced synapse loss.
- We postulate that synaptic resilience is an active neuronal program rather than a passive byproduct of pathology.

## 1 BACKGROUND

Dendritic spine integrity is a key determinant of cognitive function in Alzheimer's disease (AD). Loss of dendritic spines tightly correlates with cognitive deficits in AD patients and animal models.^1^, ^2^, ^3^ Conversely, the preservation of dendritic spines is associated with cognitive resilience—the ability to maintain normal cognition despite significant amyloid and tau pathology.^4^, ^5^, ^6^, ^7^ Notably, restoring dendritic spine density in AD animal models is sufficient to rescue cognitive function.^8^ These findings underscore the need to elucidate the compensatory and repair mechanisms that counteract spine loss in AD.^9^, ^10^

Although the mechanisms underlying dendritic spine loss are increasingly well understood, whether and how neurons compensate for spine loss remains largely unknown.^10^ Because early synapse loss in AD is gradual and localized to dendrites in close proximity to plaques,^11^, ^12^, ^13^ it is unlikely to engage classical homeostatic plasticity mechanisms like synaptic upscaling,^14^ which require sustained and widespread reductions in neuronal firing. Instead, local forms of spine compensation in the vicinity of lost spines are more likely to be implemented in the early stages of AD.

Snapshots on fixed preparations from *post mortem* AD subjects, as well as animal models of AD, have shown that dendritic spine loss co‐occurs with an enlargement of the neighboring spines.^1^, ^10^, ^15^, ^16^, ^17^, ^18^ Although this may correspond to a compensatory adaptation to preserve the excitatory drive of the affected dendrite,^1^, ^10^ this has typically been attributed to the preferential loss of small, vulnerable spines.^18^ In this view, simply increasing the proportion of large spines could account for the population‐level increase in average spine size. To distinguish between these two possibilities, it is crucial to perform longitudinal live imaging experiments to investigate whether the loss of dendritic spines leads to the emergence of compensation over time at the individual spine level.^10^

Another key challenge in studying the mechanisms of spine compensation in AD lies in the inability to predict which dendritic spines will be eliminated, and consequently, which ones will undergo compensation. To overcome this limitation, in this study we developed an optogenetic tool for the artificial elimination of dendritic spines with high spatiotemporal control and identified a two‐stage compensatory response: rapid enlargement of remaining spines followed by delayed spine regeneration. Strikingly, we observed similar structural plasticity across multiple in vitro and in vivo models of amyloid beta (Aβ)–induced synapse loss. These findings suggest that neurons retain an intrinsic capacity to reverse early synaptic loss, potentially contributing to cognitive resilience in AD.

## 2 METHODS

### 2.1 Animals

All experiments were performed under licenses approved by the UK Home Office according to the Animals (Scientific Procedures) Act and the University of Queensland Animal Ethics Committee. Female, adult Sprague–Dawley rats and their embryos (both male and female at embryonic stage 18) were used for preparing the primary hippocampal neurons. Wistar rats postnatal day 4 to 5 were used for preparing organotypic hippocampal slice cultures. Male 12‐week‐old CD1 (in utero electroporated) and C57BL/6J (AAV‐injected) mice were used for surgical procedures and in vivo imaging experiments. Animals were typically housed in groups of 6 (2–3 after surgery), in a 12 hour/12 hour light/dark cycle in standard cages containing appropriate enrichment and access to food and water.

### 2.2 Primary neuronal cultures and transient cell transfection

Primary rat hippocampal neurons were prepared from Sprague–Dawley rat embryos (males and females, embryonic day 18). Briefly, isolated hippocampi were subjected to enzymatic digestion using 10U of papain suspension (Worthington Biochemical Corporation) for 20 minutes in a 37°C water bath and further mechanically dissociated by trituration using fire polished Pasteur pipettes. The single cell suspension was then plated at a density of 8 × 10^4^ cells per dish on Poly‐l‐lysine (0.5 mg/mL; Sigma‐Aldrich) coated 20 mm glass bottom dishes (Cellvis) in plain Neurobasal plating medium (Gibco) supplemented with 5% fetal bovine serum (FBS; GE Healthcare), 1% l‐glutamine (Gibco), 1% penicillin–streptomycin (Gibco), and 2% B‐27 supplement (Gibco) and incubated in a 37°C humidified tissue culture incubator with 5% CO_2_. Cultured neurons were fed twice a week with Neurobasal medium with no serum.

Cultured hippocampal neurons were transfected between days in vitro (DIV) 11 to 14 using Lipofectamine 2000 (Invitrogen) according to the manufacturer's protocol and imaged DIV 15 onward, after a minimum 48 hours post‐transfection. Because neurons are post mitotic, exogenous plasmid DNA over expression was long lasting as well as non‐toxic. The pCMV‐Homer1c‐EGFP plasmid was obtained from the Choquet lab, Bordeaux, France. Plasmid was purified using a DNA purification kit (Qiagen) prior to transfection.

**RESEARCH IN CONTEXT**

1. **Systematic review**: The authors reviewed relevant literature using PubMed searches. Although there is strong evidence that dendritic spine loss correlates with cognitive decline in Alzheimer's disease (AD), it remains largely unknown whether and how neurons compensate for dendritic spine loss in AD. References are cited in the introduction and discussion.
2. **Interpretation**: Our findings demonstrate that dendritic spine loss in AD models triggers a two‐stage intrinsic compensatory response: initial enlargement of remaining spines followed by delayed regeneration of lost spines, challenging the prevailing notion that synapse loss in AD is unidirectional.
3. **Future directions**: The new experimental platforms introduced in this study offer a unique opportunity to investigate the molecular mechanisms of synaptic compensation and regeneration in AD. Therapeutically boosting these mechanisms may offer innovative avenues for preserving synapse integrity and thus promoting cognitive resilience to AD.

### 2.3 Organotypic slice culture preparation and biolistic transfection

Organotypic hippocampal slice cultures were prepared from postnatal day 3 to 5 Wistar rats. Hippocampal slices (400 µm thick) were prepared as described previously.^19^ Twenty‐five µg of Drebrin–KillerRed and 15 µg of enhanced green fluorescent protein (EGFP) were coated onto 12.5 mg of 1.6 µm gold beads. After 7 DIV, slice cultures were transfected by biolistic gene transfer (180 psi; Gene gun; Bio‐Rad) as previously described,^20^ allowing for a minimum 72 hours post‐transfection, before imaging.

### 2.4 Aβ oligomer preparation and pharmacological treatments

Aβ oligomer (Aβo) was generated as previously.^21^ Briefly, 1,1,1,3,3,3‐hexafluoro‐2‐propanol (HFIP)‐treated Aβ1‐42 peptide (JPT Peptide Technologies GmbH, SP‐Ab‐07_0.5) was resuspended in fresh, sterile dimethyl sulfoxide (DMSO; Sigma) as monomers, with sonication to facilitate resuspension. A 0.5 mg vial of the HFIP‐treated Aβ (1–42) peptide was dissolved in 22 µL DMSO, aliquoted, and stored at −80⁰C as a 5 mM stock solution. For oligomeric Aβ treatment, the dissolved peptide stock was diluted in F‐12 medium with l‐glutamine (Gibco) to 100 µM and then incubated overnight at 4°C. The next day, the soluble Aβ oligomers were used to treat primary cultured neurons at 2.5 µM final concentration for either 3 hours or 5 hours at 37°C. For vehicle controls, DMSO was used instead of Aβ1‐42 peptide while keeping the rest of the protocol identical and processed parallelly. The concentration of Aβ oligomers is expressed in monomer equivalents. Oligomeric nature of the Aβ preparation was confirmed using Western blots with anti‐Aβ mouse antibody 6E10 (1:2000 dilution; Covance).

Anisomycin (Tocris) and D‐AP5 (Sigma‐Aldrich) to inhibit local protein synthesis and N‐methyl‐D‐aspartate receptor (NMDAR) activity, were resuspended in fresh, sterile 100% DMSO and distilled water (UltraPure, Invitrogen) at stock concentration of 80 and 20 Mm, respectively, aliquoted, and stored at −30⁰C. Both selective inhibitors were used at a working concentration of 50 µM for the respective in vitro experiments.

### 2.5 In utero electroporation

As previously described,^22^ time‐mated CD1 pregnant dams were used at embryonic stage E15 (upper layer neurogenesis) for all experiments. Deep anesthesia of mice for this recoverable procedure was induced with an intraperitoneal (IP) injection of ketamine (100 mg/mL, Parnell Laboratories)‐xylazine (20 mg/mL, Troy Laboratories) mix at dosage of 120 mg/kg and 10 mg/kg, respectively. Full anesthesia was confirmed by toe pinch test, and the dams were placed on a heat pad. The eyes of the dams were covered with Vaseline (Unilever), to prevent drying. Before performing a laparotomy to expose the embryos from the abdominal cavity, the hair covering the abdomen was gently removed using a hair removal cream (Nair), the skin was sterilized with chlorohexidine (Pfizer), and sterile gauze (Medsure) was positioned on the skin, leaving a small circular window for the laparotomy. Once the embryos were exposed, each one of them was positioned so that the lateral telencephalic ventricles were visible through the uterine wall. Plasmid DNA with the addition of 0.0025% Fast Green dye (Sigma‐Aldrich) was then microinjected with a Picospritzer II (Parker Hannifin) using a glass pulled pipette, into the right lateral ventricle. The tip of the glass pipette (Thin Wall Glass Capillaries 1.2 mm OD / 0.90 mm ID, WPI), previously prepared using a Flaming/Brown micropipette puller (heat 495, pull 100, vel 100, Sutter Instrument Co.), was trimmed obliquely using forceps, before plasmid injection. The plasmids were then electroporated approximately into the right primary somatosensory cortex (S1) with 3 mm‐diameter microelectrodes (Nepagene) delivering five (100 ms, 1 Hz) ≈ 36 V square wave pulses from an ECM 830 electroporator (BTX Harvard Apparatus). Once this procedure was completed for each embryo, the uterine horns were replaced inside the abdominal cavity and the incision was sutured closed twice. Animals were then subcutaneously injected with 1 mL of sterile saline and recovered in a humidified chamber at ≈ 28°C. For pain relief after electroporation, an edible buprenorphine solution (0.026 mg/mL; Temgesic, Indivior) in a gel pack was positioned next to the recovering dam in the cage. The buprenorphine edible gel pack was prepared before the surgery by injecting sterile sucralose‐water gel (MediGel, ClearH2O) with 0.2 mL of edible buprenorphine solution. Dams were then monitored daily until they gave birth to live pups (at ≈ E19/P0). The presence of fluorescent patches in S1 were checked using fluorescence goggles. Only the pups with fluorescent patch were kept until they reached desired experimental age and other pups without a fluorescence patch were excluded/sacrificed.

### 2.6 Cranial window implantation

After in utero electroporation, 12‐week‐old male mice from a CD1 background were anesthetized using either isoflurane (4% for induction and 1.7%–2.5% for maintenance) or with an IP injection of a sleep mix containing 0.05 mg/mL fentanyl (Sublimaze, Piramal), 5 mg/mL midazolam (Mylan), 1 mg/mL medetomidine (ilium) in sterile saline (Mini‐Plasco, B. Braun) at a dose of 10 µL/g bodyweight before mounting them onto a stereotaxic frame (RWD Life Sciences). Body temperature was monitored and maintained at 37°C throughout the procedure. Mice were injected subcutaneously with carprofen (20 mg/kg) for preoperative analgesia and eye ointment (Lubrithal, Dechra) was applied to prevent eyes drying out. The skin over the skull was disinfected with antimicrobial cleanser (Hibiscrub) and an incision was made in the skin, which was then removed to expose the skull. A craniotomy (3–5 mm in diameter) was made above the somatosensory cortex based on the stereotaxic coordinates –1.7 mm (AP) and 2.5 mm (ML) from Bregma, formed by drilling gently using a high‐speed microdrill (RWD Life Sciences). The exposed brain was kept moist by applying sterile cortex buffer (7.3 mg/mL NaCl, 0.37 mg/mL KCl, 1.98 mg/mL D‐glucose, 2.38 mg/mL HEPES, 0.29 mg/mL CaCl2*2H2O, 0.49 mg/mL MgSO4*7H2O in MilliQ H2O, adjusted to pH 7.4) before covering with a circular glass coverslip (5 mm diameter). The window was sealed, and all the areas of exposed skull bone were covered using dental cement (Vertex Dental). A small custom‐made titanium headplate was embedded in the acrylic for head‐fixing the animal during imaging sessions.

### 2.7 Viral injection

For viral injection in 12‐week‐old male mice from a C57BL/6J background, a craniotomy was first produced as described above, before a combination of adeno‐associated viruses (AAV): AAV.hSyn.Cre.WPRE.hGH (Addgene_105553‐AAV9, diluted 1:10,000); AAV‐CAG‐FLEX‐tdTomato (RRID: Addgene_28306‐AAV9, diluted 1:10), and AAV‐FLEX‐SYN1‐EGFP‐Homer1 (custom‐made, VectorBuilder) were mixed in equal parts and injected into the somatosensory cortex (300nL) using a pulled glass micropipette and a microinjection syringe pump (RWD Life Sciences), at a rate of 100nl/minute and a depth of ≈ 500 µm below the dura. Sterile cortex buffer containing 1 mg/mL dexamethasone was applied to the exposed brain to reduce inflammation and a circular glass coverslip (5 mm diameter) was placed over the craniotomy and fixed to the skull with a cyanoacrylate glue (Power Gel, Loctite). A custom‐made steel headplate was implanted onto the exposed skull with glue and fixed with dental cement (Simplex, Kemdent). Subcutaneous injections of buprenorphine (0.1 mg/kg) and sterile saline were given for post‐operative analgesia and rehydration. Mice were placed in a heat chamber to recover and returned to their cages for 3 to 4 weeks to allow for AAV expression and clearing of inflammation under the cranial window.

### 2.8 Aβo intracortical microinjection

For Aβo microinjection mice were anesthetized with an IP injection of sleep mix containing 0.05 mg/mL fentanyl (Sublimaze, Piramal), 5 mg/mL midazolam (Mylan), 1 mg/mL medetomidine (ilium) in sterile saline (Mini‐Plasco, B. Braun) at a dose of 10 µL/g bodyweight, and after baseline in vivo two‐photon imaging were head fixed under a surgical microscope. The cement around the cranial window was removed using a high‐speed microdrill (RWD Life Sciences), before the glass coverslip was carefully lifted and removed. Mice were injected intracranially with 500nL of either Aβ oligomers (45 ng total) or vehicle DMSO (0.4%) at a rate of 200nL/minute into the cortex ≈ 500 µm (ML) from the edge of the two‐photon imaging site. A new glass coverslip was then placed over the exposed brain and fixed using dental cement (Simplex, Kemdent). A subcutaneous injection of awake mix containing 0.4 mg/mL naloxone, 0.1 mg/mL flumazenil, and 5 mg/mL atipamezole in sterile saline was given at a dose of 10ul/g bodyweight, along with sterile saline for recovery and rehydration. Mice were placed in a heat chamber to recover and returned to their cages. Subsequent two‐photon imaging sessions were carried out at 24 hours and 1 week post‐injection.

### 2.9 Microscopy

For in vitro cultured hippocampal neurons, transfected live neurons were imaged between DIV 19 and DIV 24 in an Okolab stage‐top incubator maintained at 37°C and 5% CO_2_. Before imaging, the culture medium was entirely replaced with artificial cerebrospinal fluid (ACSF; 145 mM NaCl, 2 mM CaCl_2_, 2 mM MgCl_2_, 10 mM HEPES, 10 mM D‐glucose, and 5 mM KCl in MilliQ H_2_O, adjusted to pH 7.4). Individual neurons were then imaged using a Nikon Plan Apochromat 100x/1.45 NA oil‐immersion objective on an inverted spinning disk confocal microscope (Diskovery; Andor Technology) built around a Nikon Ti‐E body (Nikon Corporation) and equipped with two Zyla 4.2 sCMOS cameras (Andor Technology) and controlled by Nikon NIS software. Confocal laser at λ488nm (1.63 mW at 10X) was used for excitation and imaging the Homer1c‐eGFP. Secondary dendrites of co‐transfected neurons were randomly imaged as z‐stack with 0.4 µm step size over a range of 5 µm at sequential time‐points. A z‐stack was captured before the Aβo or vehicle treatment (0 hours) and a second z‐stack of the same dendritic region was captured at respective time‐point (either 3 hours or 5 hours) after either treatment.

For ex vivo organotypic hippocampal slice neurons, biolistically transfected neurons were longitudinally imaged between DIV 11 and 19 in ACSF (127 mM NaCl, 2 mM CaCl_2_, 1 mM MgCl_2_, 25 mM D‐glucose, 2.5 mM KCl, 25 mM NaHCO_3_, and 1.25 mM NaH_2_PO_4_*H_2_O) maintained at the temperature of 35°C and pH 7.4. Image stacks of transfected hippocampal pyramidal neurons were acquired using two‐photon laser scanning microscopy (Ultima Multiphoton system, Bruker) equipped with a tunable Ti: Sapphire laser (Mai Tai or Chameleon Vision by Spectra Physics and Coherent, respectively) and Prairie View software (version 5.6). The image stacks (512 × 512 pixels; 0.68 µm per pixel) with 0.5 µm z‐steps were imaged using 60x immersion objective and an optical zoom of 10x, for each time‐point. For Drebrin–KillerRed inactivation, the dendritic region of interest (60 × 60 µm) was irradiated with epifluorescence wide‐field green light using a Mercury lamp (X‐Cite 120Q) until KillerRed photobleaching between 3 and 5 minutes.

For in vivo imaging, apical dendritic stretches 10 to 100 µm below the cortical surface, of co‐labelled layer II/III pyramidal neurons were repeatedly imaged in vivo in mice anesthetized with either isoflurane (4% for induction and 1.7%–2.5% for maintenance) or with an IP injection of a sleep mix (0.05 mg/mL fentanyl, 5 mg/mL midazolam, and 1 mg/mL medetomidine) at different time‐points using two‐photon laser scanning microscopy (Ultima Multiphoton system, Bruker) equipped with tunable Ti: Sapphire lasers and a x25 WI Nikon objective (NA 1.10; WD 2.0). For Drebrin–KillerRed inactivation, the dendritic region of interest was irradiated with epifluorescence wide‐field green light using a Mercury lamp (X‐Cite 120Q) for 30 minutes.

### 2.10 Image analysis

All the spine analyses were performed using FIJI (ImageJ). Unhealthy‐appearing neurons which displayed blebbing or loss of dendritic processes were excluded from analysis. Only the dendritic spines or Homer1c puncta appearing as clear protrusions adjacent to the dendrite, irrespective of their orientation relative to the imaging plane, were included in the analyses. Spine density was calculated as total number of spines or Homer1c puncta per measured dendritic length. The rate of spine loss and gain was calculated as percentages of spines that appeared and disappeared at the different time‐points after treatments, relative to the corresponding number of spines at 0 hours. The integrated fluorescence intensity (brightness) of dendritic spines (either GFP or Homer1c puncta) on the z‐stack maximal projections or the best focal section was used as a measure of spine size, as previously described.^23^ To control for differences in GFP or Homer1c‐GFP expression, as well as for variability in laser power, dendritic spine brightness was normalized to that of the adjacent dendritic shaft and corrected for background fluorescence (outside dendrite) according to the following formula: spine size = (brightness spine – background fluorescence) / (brightness dendritic shaft – background fluorescence).

Relative changes in dendritic spine or Homer1c puncta sizes were quantified as ratios of individual spine/punctum sizes following the respective manipulation to the initial size of the same spine prior to treatment. The individual values of relative change in size were then averaged per dendritic region for all treatment groups.

GCaMP analysis was based on work described previously.^24^ Briefly, to measure spontaneous spine activity regions of interest (ROIs) were drawn around spines of interest and mean gray value was measured simultaneously for all spines within the field of view for the duration of the experiment (5 minutes). Δ*F*/F was calculated by dividing the mean gray value over time for the spine (Δ*F*) with the averaged mean gray value of five ROIs drawn randomly along the dendritic shaft (F). This was done to ensure the observed changes are specific to spines and not the entire dendritic region.

### 2.11 Statistics

All statistical tests were performed using Graph Pad Prism version 8 and above. Statistical tests were chosen based on tests for normality. The specific statistical analyses performed are listed in the respective figure legends. A *p* value of < 0.05 was considered statistically significant. Depending on the experiments, either individuals’ spines (paired analysis) or neurons (independent samples) will correspond to the experimental unit. Correlation coefficients were calculated with a Spearman rank or Pearson correlation coefficient.

## 3 RESULTS

### 3.1 Optogenetic spine elimination triggers a two‐stage compensatory response

**FIGURE 1:**
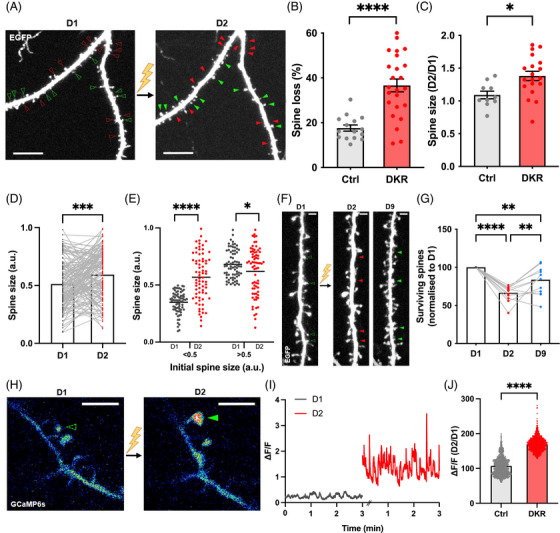
Artificial dendritic spine loss and compensation using a chromophore‐assisted light inactivation (CALI) approach. A, Two‐photon images of EGFP‐labeled dendrites in hippocampal neuron in organotypic slice cultures overexpressing GFP and Drebrin–KillerRed before and after the optical inactivation of Drebrin. Note that Drebrin inactivation leads to spine loss (red arrowheads) and spine compensation (green arrowheads) 24 hours post‐inactivation (D2) compared to baseline imaging (green/red hollow arrowheads). Scale bars 10 µm. B, Bar graph showing a significant loss of dendritic spines 24 hours post‐Drebrin inactivation. Spine loss in controls (Ctrl) = 17.6 ± 1.373%, *n *= 15 neurons; spine loss after Drebrin inactivation = 36.6 ± 2.86%. *n *= 24 neurons ****p < 0.0001 unpaired *t* test. Data points in bar graph correspond to neurons. All data are ± SEM. C, Bar graph showing a significant increase in spine size after Drebrin inactivation. Relative size increase between day 2 and day 1 (D2/D1) in control (Ctrl) = 1.091 ± 0.06 (*n* = 10 neurons) and after Drebrin inactivation (DKR) = 1.381 ± 0.07 (*n* = 19 neurons). *p < 0.05, unpaired Mann–Whitney *t* test. Data points in bar graph correspond to neurons. All data are ± SEM. D, Bar graphs showing the increase in spine size at the individual spine level after Drebrin inactivation (day 1 = 0.5133 ± 0.17; day 2 = 0.5939 ± 0.017). ***p < 0.001 paired *t* test (*n* = 143 spines). E, Graph showing that smaller dendritic spines (brightness ratio < 0.5) are preferentially enlarged whereas large spines (brightness ratio > 0.5) shrink after 24 hours. Small spines: day 1 = 0.348 ± 0.011, day 2 = 0.568 ± 0.02; ****p < 0.0001, *n* = 72. Large spines: day 1 = 0.680 ± 0.014, day 2 = 0.620 ± 0.024; *p < 0.05, *n *= 71 spines. F, Two‐photon images of hippocampal neurons showing the regrowth of dendritic spines 1 week after Drebrin–KillerRed inactivation. Note that Drebrin inactivation induces spine loss (red arrowheads) and a compensatory regrowth (green arrowheads) of lost dendritic spines (green hollow arrowheads). Scale bars 2 µm. G, Bar graph representing the regrowth of dendritic spines 1 week (day 9) post Drebrin inactivation when normalized to day 1 (day 2 = 66.59 ± 2.804 and day 9 = 83.69 ± 5.13%, day 2/day 1 ****p < 0.0001, day 9/day 1 **p < 0.01, day 9/day 2 **p < 0.01, one‐way analysis of variance, *n *= 13 neurons. H, Representative dendritic region of a neuron overexpressing the genetically encoded calcium indicator GCaMP6s and Drebrin–KillerRed. The GCaMP6s fluorescence signal in dendritic spines (green arrow) increased after Drebrin–KillerRed inactivation. I, GCaMP6s trace in the spine indicated with the green arrow in (H) before and after Drebrin–KillerRed inactivation. J, Bar graph showing a significant increase in GCaMP6s signal in individual dendritic spines 24 hours post Drebrin inactivation. Control = 107.1 ± 0.738%, DKR = 170 ± 0.552%. ****p < 0.001 unpaired *t* test (*n* = 1270 traces). EGFP, enhanced green fluorescent protein; SEM, standard error of the mean.

Because dendritic spine loss in AD occurs stochastically, it is not possible to predict which synapses will be lost or compensated. To achieve spatiotemporal control over spine loss, we developed an optical approach for the targeted elimination of spines by disrupting the actin cytoskeleton using chromophore‐assisted light inactivation (CALI).^25^ Using biolistic transfection in organotypic slices cultures, we overexpressed DrebrinA, an actin‐binding protein which is highly enriched at spines,^26^ fused to the genetically encoded photosensitizer KillerRed, along with the structural marker EGFP. After a two‐photon baseline imaging session of dendrites labeled with EGFP, we locally inactivated Drebrin–KillerRed using epifluorescent green light and reimaged the same dendritic region 24 hours and 1 week later. As depicted in Figure 1A and B, we found that Drebrin inactivation led to the artificial elimination of dendritic spines 24 hours later compared to no light controls (Drebrin–KillerRed overexpression without light inactivation) or light controls (soluble KillerRed overexpression with light inactivation; Figure S1A in supporting information). Using post hoc immunostaining of endogenous PSD95, we found that spine elimination had no effect on the density of shaft PSD95 puncta suggesting that spine synapses did not relocate onto the shaft after spine elimination (Figure S2A–C in supporting information). To investigate whether artificial spine elimination led to the enlargement of the surviving dendritic spines, we measured the size of individual spines before and after Drebrin–KillerRed inactivation by measuring their integrated EGFP brightness (see Methods). Remarkably, we found that spine elimination triggered the enlargement of the remaining spines at the individual spine level (Figure 1C and D). By plotting the extent of spine enlargement as a function of its initial size, we found that smaller spines were preferentially enlarged (Figure 1E and Figure S3A in supporting information), in agreement with their plastic nature.^27^, ^28^ Importantly, to examine whether spine enlargement was paralleled by functional changes, we artificially eliminated spines in neurons expressing Drebrin–KillerRed together with the genetically encoded calcium indicator GCaMP6s. As shown in Figure 1H–J, Drebrin inactivation led to a robust enhancement of spontaneously evoked calcium transients in individual spines 24 hours post‐inactivation.

To assess whether artificial spine elimination triggers spinogenesis as an additional form of structural compensation, we also quantified spine formation rates at 24 hours and 1 week after Drebrin inactivation. While no increase in spine formation was detected 24 hours after inactivation (Figure S3B), a substantial regeneration of dendritic spines became evident after 1 week (Figure 1F and G).

### 3.2 In vivo validation of optogenetic dendritic spine elimination and compensation

**FIGURE 2:**
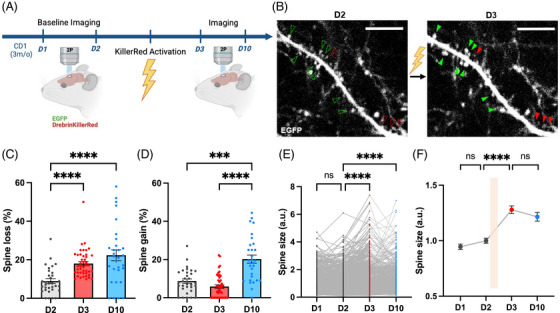
In vivo validation of optogenetic dendritic spine elimination and compensation. A, Schematic displaying the experimental workflow for in vivo two‐photon imaging and inactivation of Drebrin in wild type CD1 mouse previously in utero electroporated with Drebrin–KillerRed and EGFP. Figure was created with Biorender.com. B, In vivo two‐photon images of cortical neurons labelled with EGFP at baseline (day 2) and 24 hours post‐Drebrin inactivation (D3). Dendritic spine loss and compensation is indicated by red and green arrowheads, respectively, compared to baseline (green/red hollow arrowheads). Scale bar 20 µm. C, Bar graph showing a significant loss of dendritic spines 24 hours (day 3) and 1 week (day 10) after Drebrin inactivation in dendrites with > 10% spine loss (45/123 dendrites). Spine loss day 2 (baseline) = 8.896 ± 1.206%, *n* = 32; day 3 = 18.03 % ± 1.091, *n* = 44; day 10 = (22.34 ± 2.84%), *n* = 30. day 2 versus day 3 ****p < 0.0001, day 2 versus day 10 ****p < 0.0001, day 3 versus day 10 non‐significant Kruskal–Wallis test. D, Bar graph showing a significant increase in spine gain 1 week (day 10) after Drebrin inactivation in dendrites with > 10% spine loss (45/123 dendrites) in dendrites with > 10% spine loss (45/123 dendrites). Spine gain day 2 (baseline) = 8.705 ± 1.111%, n = 32; day 3 = 5.871 ± 0.9229%, *n *= 44 dendrites; day 10 = 20.21 ± 2.130%, *n *= 30 dendrites. Day 2 versus day 3 non‐significant, day 2 versus day 10 ***p < 0.001, day 3 versus day 10 ****p < 0.0001. Kruskal–Wallis test. E, Line graphs depicting a significant increase in the size of individual dendritic spines 24 hours (day 3) and 1 week (day 10) after Drebrin inactivation in dendrites with > 10% spine loss (45/123 dendrites). Spine sizes day 1 = 0.9457 ± 0.024 (n = 846 spines), day 2 = 1.00 ± 0.023 (*n* = 1025 spines), day 3 = 1.280 ± 0.034 (*n *= 934 spines); day 10 = 1.216 ± 0.039 (*n* = 631 spines). Day 1 versus day 2 non‐significant, day 2 versus day 3 ****p < 0.0001, day 2 versus day 10 ****p < 0.0001, day 3 versus day 10 non‐significant Kruskal–Wallis test. F, Line graph depicting the averaged spine size for data showed in (E). Note that spine loss mediated by Drebrin inactivation (orange bar) leads to the long‐term enlargement of the remaining dendritic spines. ****p < 0.0001. EGFP, enhanced green fluorescent protein.

Finally, we evaluated whether artificial spine elimination induces structural compensation in vivo. To that end, we delivered the optogenetic tool and the structural marker GFP in the cortex via in utero electroporation at E15 and implanted a cranial window postnatally to allow for two‐photon imaging and Drebrin inactivation (Figure 2A,B). After two consecutive baseline imaging sessions (day 1 and day 2), we locally inactivated Drebrin–KillerRed using epifluorescent green light and reimaged the same dendritic region 24 hours and 1 week later using in vivo two‐photon imaging. Although artificial spine elimination was less effective in vivo than in vitro—likely due to limited brain penetration of one‐photon light—dendrites with > 10% spine loss (Figure 2C) showed enlargement of surviving spines at 24 hours (Figure 2E,F) and increased spine formation after 1 week (Figure 2D).

### 3.3 Aßo‐mediated spine elimination triggers a two‐stage compensatory response

**FIGURE 3:**
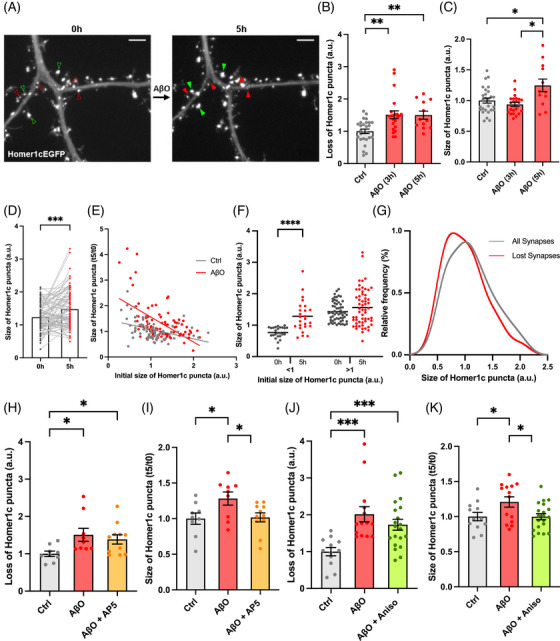
Mechanisms of spine compensation in an in vitro Alzheimer's disease model. A, Representative dendritic region of a primary cultured hippocampal neuron transfected with Homer1c‐EGFP before (left) and after (right) 5 hours of Aβo treatment. Note that Aβo induces loss of dendritic spines (red arrowheads) and compensatory enlargement of dendritic spines in remaining synapses (green arrowheads) compared to baseline imaging (red and green hollow arrowheads). Scale bars 3 µm. B, Bar graph showing a significant loss of Homer1c puncta at both 3 and 5 hours of Aβo treatment (3 hours: 19.82% ± 1.562 with a relative loss = 1.511; 5 hours: 24.37% ± 2.015; relative loss = 1.5) compared to the vehicle treated (DMSO) controls (13.11% ± 1.194 and 16.25% ± 1.836 at 3 and 5 hours, respectively; pooled data). **p < 0.01, unpaired Kolmogorov–Smirnov test and unpaired *t* test with Welch correction respectively. Data points in bar graph corresponds to neurons. All data are ± SEM. C, Bar graphs representing the size of Homer1c puncta in surviving spines before (Ctrl; pooled for 3 and 5 hours) and after 3 hours (relative size 0.9366 ± 0.0323) or 5 hours (1.246 ± 0.1030) of Aβo treatment, respectively. *p < 0.05, unpaired *t* test with Welch correction. Data points in bar graph corresponds to neurons. All data are ± SEM. D, Bar graphs representing the increase in size of Homer1c puncta at the individual spine level before (1.230 ± 0.0454 a.u.) and after 5 hours (1.476 ± 0.06302 a.u.) of Aβo treatment respectively. ***p < 0.001, paired Wilcoxon test. *n *= 83 spines from 12 neurons. E, Significant negative correlation observed between the initial size of Homer1c puncta at *t *= 0 hours and its relative size after 5 hours in both Aβo‐treated (*r* = –0.4244) and vehicle controls (*r* = –0.4393). ****p < 0.0001, Spearman correlation test. Simple linear regression analysis shows that slopes are significantly different (**p < 0.01) between the two treatment groups. F, Graph showing that Homer1c puncta in smaller dendritic spines (brightness ratio < 1) are significantly enlarged after 5 hours, with no change for larger spines (brightness ratio > 1). ****p < 0.0001, paired Wilcoxon test. Data points in bar graph correspond to individual puncta. G, Frequency distribution for the size of lost Homer1c puncta compared to the size of all Homer1c puncta in the original population. Note that the distribution of the lost synapses is slightly shifted to the left indicating a preferential loss of smaller synapses. H, Bar graph showing a significant loss of Homer1c puncta in both Aβo only (positive control; 1.508 ± 0.1726, *n *= 9) and C + D‐AP5 (1.385 ± 0.1249, *n* = 11) treated neurons after 5 hours, relative to controls (*n* = 9). Note that the controls represent the loss of Homer1c puncta in neurons after 5 hours incubation with the vehicle DMSO co‐treated in the last 2 hours with D‐AP5 (without Aβo). *p < 0.05, unpaired *t* test with Welch correction. All data are ± SEM. I, Bar graphs showing that the relative size of Homer1c puncta in surviving spines was enlarged only in positive‐control Aβo only treatment (1.282 ± 0.0916) but not in Aβo + D‐AP5 (1.017 ± 0.0635) or controls. Note that the controls represent the relative size of Homer1c puncta in spines after 5 hours incubation with the vehicle DMSO co‐treated in the last 2 hours with D‐AP5 (without Aβo). *p < 0.05, unpaired *t* test with Welch correction. All data are ± SEM. J, Bar graph showing that the loss of Homer1c puncta was induced in both positive‐control Aβo only (2.014 ± 0.2061, *n* = 14) and Aβo + anisomycin (1.730 ± 0.1485, *n* = 20) treated neurons after 5 hours, respectively, relative to controls (*n* = 12). Note that the controls represent the loss of Homer1c puncta in neurons after 5 hours of incubation with the vehicle DMSO and anisomycin (without Aβo). ***p < 0.001, unpaired *t* test with Welch correction and unpaired Kolmogorov–Smirnov test, respectively. All data are ± SEM. K, Bar graphs showing that the relative size of Homer1c puncta in surviving spines was significantly enlarged only in positive‐control Aβo only treatment (1.208 ± 0.0729) but not in Aβo + anisomycin (0.9986 ± 0.0434) or controls. Note that the controls represent the relative size of Homer1c puncta in spines after 5 hours of incubation with the vehicle DMSO and anisomycin (without Aβo). *p < 0.05, unpaired *t* test with Welch correction. *n* values represent the number of neurons. All data are ± SEM. Aβo, amyloid beta oligomer; DMSO, dimethyl sulfoxide; EGFP, enhanced green fluorescent protein; SEM, standard error of the mean.

To examine whether these forms of compensation are relevant to AD, we first implemented an in vitro model of Aβ‐induced synapse loss^29^ to track the emergence of spine compensation. Using confocal longitudinal imaging, we visualized dendritic regions before and after 3 and 5 hours of acute Aßo treatment in primary cultured neurons overexpressing Homer1c_GFP. We found Aßo triggered comparable levels of dendritic spine loss both at 3 and 5 hours (Figure 3A and B). To investigate whether this was accompanied by the enlargement of the surviving dendritic spines, we measured the size of individual spines before and after Aßo application. Strikingly, we found a significant enlargement of remaining dendritic spines after 5 but not 3 hours of treatment (Figure 3C and D) indicating that structural compensation does not occur concurrently with spine loss but emerges at a later stage. Similar to optogenetic spine elimination, we found that smaller spines were selectively enlarged after 5 hours of treatment with Aßo (Figure 3E–F). Consistent with previous studies,^15^, ^17^ we found that although Aβo induced dendritic spine loss across the entire size distribution, small spines were slightly more susceptible at both 3 and 5 hours (Figure 3G and Figure S4A in supporting information).

To begin investigating the mechanisms underlying spine compensation, we first examined the role of NMDARs. Because we and others have previously shown that Aßo‐mediated spine loss also depends on NMDAR activation,^29^, ^30^, ^31^ we applied the pharmacological inhibitor AP5 (50 µM) during the final 2 hours of the 5 hour Aßo incubation—when spine loss had already occurred and spine enlargement was likely taking place. As shown in Figure 3H and I, AP5 blocked dendritic spine enlargement, indicating that NMDAR activation contributes to the compensatory response.

Given the delayed nature of compensation, we next examined the role of new protein synthesis. Co‐incubation of Aßo with anisomycin throughout the 5 hour incubation revealed that while new protein synthesis is not required for Aßo‐mediated spine loss, it is essential for the compensatory enlargement of dendritic spines (Figure 3J, K).

Likely due to the acute nature of the treatment, we failed to detect an increase in synaptogenesis at either 3 or 5 hours (Figure S5A and B in supporting information), and assessment at 24 hours was precluded by reduced neuronal viability.

### 3.4 In vivo validation of Aßo‐mediated spine elimination and compensation

**FIGURE 4:**
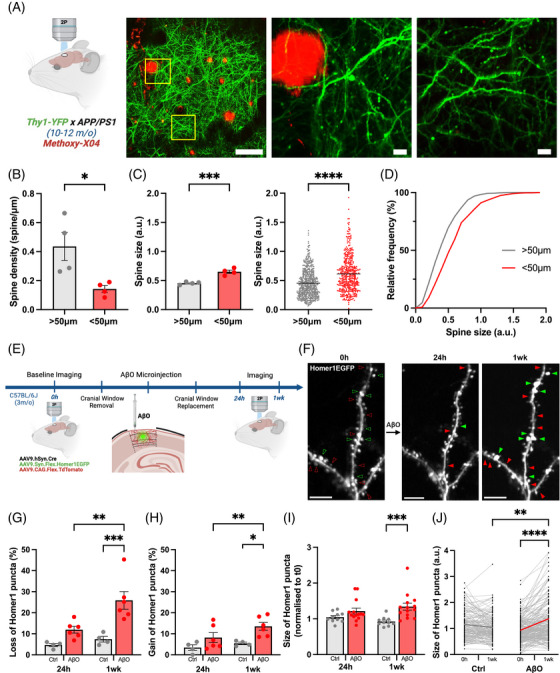
In vivo dendritic spine compensation in Alzheimer's disease models. A, Schematic (left) and images (right) showing the in vivo two‐photon imaging of YFP‐labeled dendrites in proximity of methoxy‐X04 labelled Aβ plaques in the APP/PS1xThy1‐YFP double transgenic mice. Scale bars 100 µm (left image) and 10 µm (middle and right image). Figure was created with Biorender.com. B, Bar graphs showing significant decrease in spine density within 50 µm of Aβ plaques (0.1424 ± 0.02359, *n* = 4 mice) compared to spines > 50 µm (0.4358 ± 0.09705, *n *= 4 mice). Unpaired *t* test, *p < 0.05. All data are ± SEM. Figure was created with Biorender.com. C, Graphs showing a significant enhancement in spine size within 50 µm of Aβ plaques at the level of both the animal (left; < 50 µm = 0.6508 ± 0.02870, > 50 µm = 0.4543 ± 0.01237, unpaired *t* test, *n *= 4 mice, ***p < 0.001) and individual dendritic spines (right; < 50 µm = 0.6148 ± 0.01473, n = 438; > 50 µm = 0.4538 ± 0.00999, *n *= 561; unpaired *t* test, ****p < 0.0001). Note that spine size analysis was conducted in same dendrites as in (B). All data are ± SEM. D, Cumulative distribution of dendritic spines sizes in proximity (< 50 µm) or farther away (> 50 µm) from methoxy‐X04 labeled Aβ plaques. E, Schematic displaying experimental workflow involving longitudinal in vivo two‐photon imaging before and after intra‐cortical microinjection of oligomeric forms of the Aβ peptide (Aßo) in anesthetized mice. F, In vivo two‐photon images of EGFP‐Homer1c labelled dendrites in the cortex before and after intra‐cortical Aßo injection. Note that Aßo induces dendritic spine loss (red arrowheads) and dendritic spines compensation—either enlargement of surviving spines or gain of new spines (green arrowheads)—compared to baseline imaging (red and green hollow arrowheads). Scale bars 10 µm. G, Bar graphs showing higher loss of Homer1c puncta at 24 hours and 1 week post‐injection of Aßo compared to DMSO vehicle controls (24 hours: Ctrl 4.675 ± 0.7728, Aßo 11.93 ± 1.589; 1 week: Ctrl 7.450 ± 1.333, Aßo 25.88 ± 4.178). Two‐way ANOVA with Fisher LSD, *n *= 4/6 mice, 2 to 4 cells averaged per mouse, **p < 0.01, ***p < 0.001. All data are ± SEM. H, Bar graphs showing higher gain of Homer1c puncta at 24 hours and 1 week post‐injection of Aßo compared to DMSO vehicle controls (24 hours: Ctrl 3.425 ± 1.349, Aßo 8.200 ± 2.351; 1 week: Ctrl 5.425 ± 0.5935, Aßo 13.52 ± 1.864). Two‐way ANOVA with Fisher LSD, n = 4/6 mice, 2 to 4 cells averaged per mouse, *p < 0.05, **p < 0.01. All data are ± SEM. I, Relative size of Homer1 puncta demonstrates a significant increase at 1 week post‐injection in Aßo treated dendrites compared to DMSO controls (24 hours: Ctrl 1.045 ± 0.04580, Aßo 1.223 ± 0.08846; 1 week: Ctrl 0.9255 ± 0.04098, Aßo 1.341 ± 0.09895). Two‐way ANOVA with Fisher LSD, *n *= 10/14 dendrites, ***p < 0.001. All data are ± SEM. J, Line graphs depicting a significant increase in the size of individual dendritic spines in Aßo treated mice after 1 week, compared to DMSO controls (Ctrl: 24 hours: 1.116 ± 0.04277, 1 week 1.032 ± 0.04050; Aßo: 24 hours: 0.9270 ± 0.03360, 1 week 1.213 ± 0.05368). Two‐way ANOVA with Tukey, *n* b  148/195 spines, **p < 0.01, ****p < 0.0001). All data are ± SEM. Aβ, amyloid beta; ANOVA, analysis of variance; DMSO, dimethyl sulfoxide; EGFP, enhanced green fluorescent protein; LSD, least significant difference; SEM, standard error of the mean; YFP, yellow fluorescent protein.

Last, we investigated whether spine compensation occurs in in vivo models of Aβ‐induced synapse loss. We first verified that the dendritic spine loss previously reported in the APP/PS1 model of AD ^16^, ^17^, ^32^, is accompanied by enlargement of the remaining spines. As dendritic spine loss only occurred in close proximity to amyloid plaques (< 50 µm),^11^, ^12^, ^33^ we explored whether enlargement of remaining spines is also restricted to this region. Using in vivo two‐photon imaging to simultaneously image yellow fluorescent protein (YFP)‐labeled dendrites and methoxy‐X04‐labeled plaques in double transgenic APP/PS1xThy1‐YFP mice (Figure 4A), we confirmed that dendrites close to plaques not only have a reduction in spine density, but also show significantly enlarged spines (Figure 4A–D). Because amyloid plaque deposition is stochastic, thus precluding the acquisition of baseline imaging prior to plaque deposition, it remained unclear whether the enlargement of the remaining spines originated from the loss of dendritic spines. To circumvent this issue, we longitudinally imaged the same dendritic region before and after the local injection of Aßo in the cortex of wild type C57BL/6 mice. We used an AAV‐driven Cre‐lox system (AAV9.hSyn.Cre, AAV9.flex.hSyn.EGFP‐Homer1c, and AAV9.CAG.flex_tdTomato) to sparsely express Homer1c and tdTomato—synaptic and structural markers, respectively. After baseline imaging, we injected Aßo directly into the cortex and imaged the same dendritic region 24 hours and 1 week later (Figure 4E and Figure S6A in supporting information). As expected, we found that Aßo triggered loss of dendritic spines at both time‐points, though it only reached significance after 1 week (Figure 4F and G). We then measured the size of individual spines before and after Aßo injection and found that retained dendritic spines were significantly enlarged 1 week post‐treatment at both the synaptic (Homer1cGFP; Figure 4I and J) and structural (dtTomato; Figure S6B–F) level. Critically, injection of vehicle control failed to induce dendritic spine loss or structural compensation (Figure 4G–J).

We then explored whether Aßo‐mediated spine loss is followed by an enhancement in the formation of new dendritic spines. In agreement with the artificial system, we found that the loss of spines was followed by a significant increase in new spine formation at 1 week compared to vehicle control (Figure 4H), suggesting activation of compensatory spinogenesis mechanisms to counteract dendritic spine loss.

## 4 DISCUSSION

Here, we developed an optical tool for the selective elimination of dendritic spines to model this key feature of AD and identified a two‐stage compensatory response: a rapid enlargement of surviving spines followed by delayed spine regeneration. More importantly, we showed that these structural adaptations also occur in AD models suggesting that neurons retain an intrinsic capacity to reverse early synaptic loss in AD, potentially contributing to cognitive resilience.

The integrity of dendritic spines plays a critical role in sustaining cognitive function in AD. Numerous studies have shown that spine loss strongly correlates with the severity of cognitive impairment in both patients and animal models.^1^, ^2^, ^8^, ^34^ Conversely, the preservation of dendritic spines is associated with cognitive resilience—the ability to maintain normal cognition despite significant amyloid and tau pathology.^4^, ^5^, ^7^ Despite the central importance of dendritic spines in AD, the neuronal mechanisms that compensate for their loss remain largely unknown.^10^ Given that early synapse loss in AD is gradual and localized to dendrites in close proximity to plaques,^11^, ^12^, ^13^ it is unlikely to engage classical homeostatic plasticity mechanisms like synaptic upscaling, which require sustained and widespread reductions in neuronal activity.^14^ Instead, local forms of structural compensation like the ones identified in this study are more likely to be implemented in the early stages of AD.

Although *post mortem* studies of AD patients and animal models have reported that dendritic spine loss is often accompanied by enlargement of the remaining spines,^1^, ^10^, ^15^, ^16^, ^17^, ^18^ this has typically been attributed to the preferential loss of small, vulnerable spines.^18^ As such, the apparent increase in average spine size would simply reflect the surviving population of larger spines. However, by using longitudinal imaging of the same dendritic regions over time, we provide direct evidence that the spine enlargement observed in AD reflects a genuine compensatory response at the level of an individual spine. First, we established a causal link between spine loss—induced either by our artificial tool or by Aβo—and the compensatory enlargement of pre‐existing spines at the single‐spine level. Second, although Aβo preferentially targeted small spines, this alone could not account for the population‐level increase in spine size. Notably, a 3 hour incubation with Aβo was sufficient to induce spine loss but did not result in population‐wide spine enlargement. Interestingly, we observed that small spines were selectively enlarged—consistent with their higher plasticity^27^, ^28^—which further decreased their relative representation at the population level. Last, previous correlative light and electron microscopy (CLEM) studies have demonstrated a strong correlation between two‐photon measurements of spine volume (via GFP brightness) and the post‐synaptic density size,^23^, ^35^ indicating that the compensatory enlargement of surviving spines is associated with functional changes in synaptic transmission. This is further supported by our findings showing that compensated spines show increased calcium responses.

In addition to demonstrating that spine enlargement represents a bona fide form of structural compensation, we also provide the first mechanistic insight into this process. Specifically, we show that compensatory spine enlargement depends on NMDAR activation and *de novo* protein synthesis. While further studies are needed to fully characterize this form of compensation, the involvement of NMDARs suggests that active spines are preferentially engaged in this compensatory response, supporting its physiological relevance. Moreover, the established role of NMDARs in driving calcium‐dependent gene expression aligns with the requirement for new protein synthesis in this form of structural plasticity.^36^, ^37^

Another potential mechanism of compensation for spine loss in AD is the *de novo* formation of dendritic spines. This regenerative response may represent a more robust form of structural compensation than the enlargement of existing spines, as it has the potential to restore overall spine density and re‐establish lost synaptic connectivity.^10^ Although previous studies using Aβ models of AD have provided indirect evidence of increased synaptogenesis,^10^, ^38^, ^39^ it remained unclear whether this represented a true compensatory response or was simply a consequence of APP overexpression, which is known to promote synaptogenesis during development.^40^, ^41^ In this study, we provide direct evidence that both Aβo and optogenetically induced spine loss drive *de novo* spine formation in wild‐type animals, establishing a causal link between synapse loss and compensatory spinogenesis. While these findings support the idea that neurons retain the capacity to reverse early synaptic loss in AD, it remains to be determined whether the newly formed spines successfully re‐establish functional synaptic connectivity.

A key question emerging from this study is whether the observed structural changes contribute to cognitive resilience—the capacity to maintain normal cognitive function despite substantial amyloid and tau pathology.^42^, ^43^ Although small spines are generally considered “learning” spines and associated with memory,^28^, ^44^ a recent study in the oldest old (mean age ≈ 90 years) found that larger spines in the temporal cortex better predict episodic memory performance,^45^ potentially reflecting the cognitive benefits of the compensatory enlargement of dendritic spines.

While previous studies have shown that cognitively resilient individuals retain dendritic spine densities similar to age‐matched controls,^4^, ^5^, ^7^ the mechanisms underlying this preservation remain unclear. Our findings suggest that an enhanced capacity for spinogenesis could play a role in counteracting spine loss in these individuals. Alternatively, resilience may arise from a higher baseline spine density or from reduced vulnerability of their spines to Aβo—for instance, due to the absence of critical Aβo‐binding receptors. Notably, cognitive resilience has been associated with an increased proportion of thin, elongated spines,^4^, ^7^ which may represent newly formed spines actively engaging in synaptogenesis.^23^, ^46^ This hypothesis is further supported by gene ontology analyses from multiple transcriptomic and proteomic studies of resilient individuals, which show enrichment in pathways related to cell–cell adhesion—processes known to be essential for synaptogenesis during development.^47^, ^48^, ^49^, ^50^

In conclusion, we identified a two‐stage compensatory response to dendritic spine loss: rapid enlargement of surviving spines followed by delayed spine regeneration. As these structural adaptations also occur in models of AD, our spine compensation framework offers a powerful platform to uncover fundamental molecular mechanisms that could be leveraged to reverse early synaptic loss and enhance cognitive resilience in AD.

## CONFLICT OF INTEREST STATEMENT

All authors declare no conflicts of interest. Author disclosures are available in the supporting information.

## CONSENT STATEMENT

Consent was not necessary.

## Supporting information



Supporting Information

Supporting Information
